# A transcriptional plasticity-aware framework for RNA-seq differential expression analysis

**DOI:** 10.1093/bib/bbaf557

**Published:** 2025-10-20

**Authors:** Cheng Bei, Xiaoman Wang, Mingyu Gan, Howard E Takiff, Eric J Rubin, Junhao Zhu, Qian Gao, Qingyun Liu

**Affiliations:** Key Laboratory of Medical Molecular Virology (MOE/NHC/CAMS), School of Basic Medical Science, Shanghai Medical College, Shanghai Institute of Infectious Disease and Biosecurity, Fudan University, 138 Yixueyuan Rd, Xuhui District, Shanghai 200032, China; Laboratory of Pathogen Microbiology and Immunology, Institute of Microbiology, Chinese Academy of Sciences, No. 1 Beichen West Road, Chaoyang District, Beijing 100101, China; Department of Genetics, University of North Carolina at Chapel Hill, 120 Mason Farm Rd, Chapel Hill, NC 27599, United States; Instituto Venezolano de Investigaciones Cientificas (IVIC), Carr. Panamericana, Parroquia Macarao 1204, Miranda, Venezuela; Department of Immunology and Infectious Diseases, Harvard T. H. Chan School of Public Health, 665 Huntington Avenue, Boston, MA, United States; Laboratory of Pathogen Microbiology and Immunology, Institute of Microbiology, Chinese Academy of Sciences, No. 1 Beichen West Road, Chaoyang District, Beijing 100101, China; Key Laboratory of Medical Molecular Virology (MOE/NHC/CAMS), School of Basic Medical Science, Shanghai Medical College, Shanghai Institute of Infectious Disease and Biosecurity, Fudan University, 138 Yixueyuan Rd, Xuhui District, Shanghai 200032, China; Department of Genetics, University of North Carolina at Chapel Hill, 120 Mason Farm Rd, Chapel Hill, NC 27599, United States; Department of Microbiology and Immunology, University of North Carolina at Chapel Hill, 125 Mason Farm Rd, Chapel Hill, NC 27599, United States

**Keywords:** RNA-seq, differential expression analysis, bacteria, mycobacterium, transcriptional plasticity

## Abstract

Differential expression (DE) analysis based on transcriptomic data provides a genome-wide assessment of gene responsiveness. We recently characterized transcriptional plasticity (TP)—the variability of gene expression in response to environmental changes—but its impact on DE analysis remained unexplored. In this work, we examined how TP affects DE analysis and introduced a TP-aware framework to improve the interpretation of DE results. We revealed correlations between fold change of gene expression and TP in 238 experiments with Mycobacterium tuberculosis (*Mtb*) and Escherichia coli (E. coli), which carried inherent biases, favoring genes with high TP while overlooking those with low TP. Therefore, we employed Locally Estimated Scatterplot Smoothing on TP to adjust the fold change of gene expression. Adjusted DE analyses identified new responsive pathways and yielded higher overall statistical significance and enrichment scores, especially for pathways with low-TP genes. Specifically, adjusted DE results revealed that bedaquiline treatment of *Mtb* induced cholesterol degradation, linezolid repressed acetate metabolism, and infection of macrophages upregulated fatty acid metabolism while downregulating cofactor biosynthesis. We also demonstrate that the adjustment strategy can be applied to other bacterial species and is compatible with various RNA-seq quantification approaches. In summary, we introduce a TP-aware approach that normalizes DE analysis by correcting for inherent transcriptional variability.

## Introduction

Differential expression (DE) analysis of transcriptomic data provides a genome-wide assessment of changes in mRNA expression, while downstream enrichment analysis identifies differentially expressed genes (DEGs) whose mRNA levels vary significantly under specific experimental conditions [1–3]. Typically, a single DE analysis can yield tens to over a thousand DEGs, making it difficult to prioritize the important response pathways. Therefore, genes are often filtered based on their levels of DE and interest is concentrated on those with changes exceeding an arbitrary threshold [e.g. a 2-fold change (FC)] [4–6]. Alternatively, rank-based strategies, such as Gene Set Enrichment Analysis (GSEA), allow for a comprehensive enrichment based on ranking the DE levels (i.e. fold change of gene expression) of all DEGs, thereby providing a more robust measure of the responses [7, 8]. In DE analysis approaches, however, there is a consensus that the biological effect depends, at least partially, on the magnitude of the variation in gene expression [9], and therefore genes exhibiting the largest FC in mRNA abundance are more likely to be prioritized in downstream analyses [10].

However, the biological impact of the magnitude of change in the level of expression varies across genes, and for some genes even small changes in expression can be biologically important [6, 11–13]. For example, Hughes *et al.* subjected Saccharomyces cerevisiae to various genetic and chemical treatments and found that genes in certain functional categories (e.g. protein synthesis) exhibited very low-magnitude of transcriptional regulation [11]. Recent studies have also suggested that the magnitude of the change in mRNA expression in response to different conditions can be an inherent property of a gene [14–17]. For each gene, gene expression level and the range of variation in gene expression are dependent upon various genetic determinants, including its promoter sequences and sites for binding ﻿RNA polymerases and specific regulatory proteins [14, 16]. Some genes have a wide range of expression levels, whereas other genes have a more restricted amplitude of variation in their levels of expression. Therefore, by defining *a priori* the range of DE for each gene, the biological significance of changes in their expression can be more accurately assessed.

A practical approach to estimating the *a priori* range of DE is to analyze transcriptomic profiles across a wide range of conditions to identify genes with frequent and wide fluctuations in expression and those with only modest changes in their level of expression. These mRNA dynamics illustrate the concept of transcriptional plasticity (TP), which quantifies the range of variation in gene expression across different conditions [15, 18, 19]. Here, we propose a refined strategy for DE analysis i.e. adjusted to the inherent TP of each gene.

In our previous work, we performed a genome-wide quantification of the TP of Mycobacterium species [15] to provide an empirical measurement of transcriptional expression variability for each gene. In the current study, we incorporated TP into DE analysis as an adjustment factor. When we normalized DE levels based on the inherent TP of each gene, we observed increased statistical significance and pathway enrichment scores, indicating improved detection of bacterial responses. We then applied the TP adjustment to newly generated RNA-seq data from *Mtb* and two other bacterial species—Bacillus subtilis (B. subtilis) and Pseudomonas aeruginosa (P. aeruginosa) —to demonstrate that the strategy can be used to enhance the interpretability of transcriptomic data obtained with different DE analysis methods and from different species.

## Results

### TP acts as an *a priori* indicator for DE range

**Figure 1:**
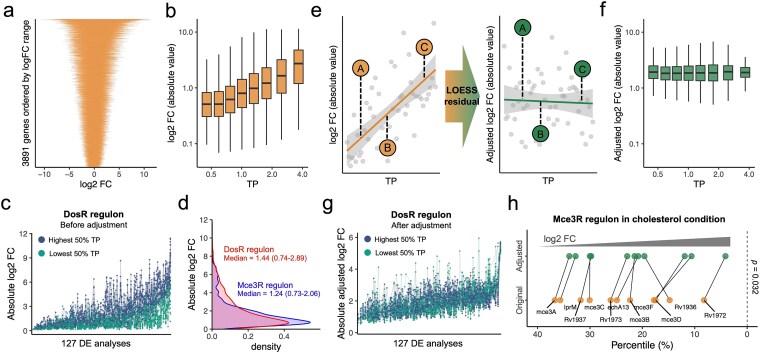
Adjusting differential expression (DE) levels using transcriptional plasticity (TP) in *Mtb*. (a) Distribution of log₂ fold change (FC) values for 3891 *Mtb* genes, ordered by their log₂ FC range. Each line represents the log₂ FC range of an individual gene across 127 DE analyses. (b) Absolute log₂ FC values are positively correlated with TP among 294 140 differentially expressed genes (DEGs) with an adjusted *P* < .05 across 127 DE analyses of *Mtb* RNA-seq datasets. DEGs are grouped into eight bins with equal logarithmic TP ranges. Each box represents a bin, with boxes showing the median and interquartile range (IQR) of log₂ FC, while whiskers represent the median ± 1.5 × IQR. (c) Absolute log₂ FC of DosR regulon genes across 127 paired DE analyses in *Mtb* RNA-seq datasets. Experiments are ordered by the median absolute log₂ FC of DosR regulon genes. Blue dots represent genes in the highest 50% TP group within the DosR regulon, while green dots represent genes in the lowest 50% TP group. (d) Distribution of absolute log₂ FC values for DosR (red) and Mce3R (blue) regulon genes across 127 DE analyses of *Mtb* RNA-seq datasets. Median and IQR of absolute log₂ FC for each regulon are shown. (e) Schematic diagram illustrating the adjustment of DE levels using TP. Solid lines represent LOESS fits of log₂ FC (orange) and adjusted log₂ FC (green) on TP. Colored dots represent three genes (A, B, and C), where gene C exhibited the highest absolute log₂ FC, followed by genes a and B, but after TP adjustment gene a exhibited the highest absolute log₂ FC because it had highest LOESS residual. Dashed vertical lines denote LOESS residuals. (f) Absolute adjusted log₂ FC values are no longer correlated with TP among 294 140 *Mtb* DEGs. DEGs are grouped into eight bins with equal logarithmic TP ranges. Each box represents a bin, with boxes showing the median and IQR of log₂ FC, while whiskers represent the median ± 1.5 × IQR. (g) Absolute adjusted log₂ FC values of DosR regulon genes across 127 paired DE analyses in *Mtb* RNA-seq datasets. Experiments are ordered by the median absolute adjusted log₂ FC. Blue dots represent genes in the highest 50% TP group within the DosR regulon, while green dots represent genes in the lowest 50% TP group. (h) Rank percentile of DE levels for Mce3R genes significantly shifted forward among the total 2778 DEGs after adjustment. A lower percentile value indicates a higher log₂ FC. The paired Wilcoxon *P*-value is shown.

To estimate the expected DE range for bacterial genes, we curated a comprehensive RNA-seq dataset from *Mtb* strain H37Rv, including 127 DE analyses performed under various experimental conditions (Table S1). We also curated an integrated dataset from E. coli strain MG1655 obtained from the iModulon database [20] that included 111 paired experiments (Table S1). DEGs and their FC values were identified with the ﻿Linear Modeling for Microarray Data (limma) method (Data S1 and S2, Materials and Methods). In experimental conditions, the range of log₂ FC in the levels of expression varied greatly for different *Mtb* genes (Fig. 1a). For example, the range of log₂ FC for *pepQ* was only −0.64–1.08, while for *tgs1* the log₂ FC spanned from −8.23 to 11.31. Therefore, a log₂ FC of 1 would indicate maximum DE for *pepQ* but only a small DE for *tgs1*. Similar observations in E. coli (Fig. S1a), suggest that variations in the range of gene expression exist across different bacterial species.

We posited that each gene’s TP, obtained from large-scaled transcriptomic datasets, can serve as a proxy for the amplitude of the gene’s DE. In our previous study [15], the TP of *Mtb* genes was quantified by the mean-adjusted standard deviation of the trimmed mean of M-values normalized to reads per kilobase million (RPKM) from over 800 RNA-seq samples (Table S2, Materials and Methods), and thus represents the variability of transcriptional expression levels. In the current study, we used the same approach to measure the TP of E. coli genes (Table S2, Materials and Methods). In both *Mtb* and E. coli, DE levels were positively correlated with their TP, such that genes with higher TP exhibited greater FC in their levels of expression (Fig. 1b, Fig. S1b) and were more likely to exceed the commonly used DE threshold of 2-FC (Figs S1c–d). We also analyzed three newly published *Mtb* RNA-seq datasets, including strains deleted for genes encoding cysteine desulfurase (*iscS*) [21], or a metalloprotease (*rip1*) [22], and strains exposed to *Artemisia afra* extract [23]. Consistently, the DE levels reported in the published studies showed a significant and robust correlation with the TP across these different datasets, independent of the RNA-seq processing strategies (Figs S1e–g).

### Adjust DE levels using TP

We hypothesized that adjusting the degree of a gene’s DE based on its TP would improve the sensitivity of DE analysis. Bacterial genes that are co-regulated in response to different conditions are grouped into functional units termed regulons [24–26], but the genes within a regulon can exhibit varying levels of TP [15]. For example, different genes in the hypoxia-responsive DosR regulon show marked variation in their DE changes (Fig. 1c, S2a), and genes with high-TP are more frequently candidates for further study, while low-TP genes may be overlooked (Fig. S1c and d). However, the TP appears to be linked to the biological function of the gene: genes with low-amplitude expression changes were involved in protein synthesis in *S. cerevisiae* [11], and in our previous work, we found that the functions of high-TP genes were enriched in stress responses, while essential genes exhibited lower TP [15]. The genes in some regulons, such as the Mce3R regulon involved in lipid metabolism, exhibit low TP levels (Fig. S2a and b) [15, 27, 28] and were less likely to be identified as DEGs compared to genes in high TP regulons such as DosR (Fig. S2b and c, Fig. 1d). This suggests that standard DE analysis may overlook lipid metabolism responses due to the lower variability of expression of the Mce3R genes.

To adjust DE levels, we applied locally estimated scatterplot smoothing (LOESS) regression to model the relationship between FC in expression and TP. LOESS is a nonparametric approach that captures nonlinear relationships without assuming a specific functional form [29], making it well-suited for modeling the complex dependency between TP and DE levels. The residuals from this regression were then added to the average FC to obtain adjusted DE levels (Fig. 1e, Data S1, Materials and Methods), which had higher medians but reduced variability compared to the original distribution (Fig. S3a and b). Importantly, the adjusted DE levels were no longer correlated with TP (Fig. 1f, Figs S3c–e), indicating that the influence of TP on DE levels had been effectively reduced.

We hypothesized that the adjusted DE levels would better capture concordant gene responses within a regulon, leading to a more robust interpretation of DE levels across genes. After adjusting the DEs for the DosR regulon (Fig. S2a), we observed that co-regulated genes in the lowest 50% TP group exhibited DE levels comparable to those in the highest 50% TP group (Fig. 1g). The adjustment also led to a decrease in the coefficient of variation (CoV) of DE levels across the genes in the DosR regulon (Fig. S3f). Furthermore, after adjustment, all of the 35 regulons we analyzed exhibited significant decreases in the CoV of the DE levels, (Fig. S3g, Table S3, Materials and Methods), suggesting more concordant gene responses than were apparent before adjustment. To assess whether genes in low-TP regulons could be more prominently identified, we analyzed an RNA-seq dataset generated in the presence of cholesterol [30], After adjusting the DE levels for the low TP genes in the Mce3R regulon (Fig. S2b), the ranking of 11 Mce3R regulon genes improved by an average of 70.4 positions among the total 2778 DEGs (Fig. 1h). To test whether the improved ranking would benefit capture of Mce3R regulon response in DE analysis, we applied GSEA [7] (Materials and Methods) to the cholesterol dataset [30]. Notably, the enrichment significance of Mce3R regulon improved substantially after TP adjustment: the adjusted *P*-value decreased from 0.11 to 0.00096, and the normalized enrichment score (NES) increased from 1.67 to 2.25 (Fig. S3h). To assess whether this effect was specific to the Mce3R regulon, we performed the same GSEA analysis across all other known regulons in our dataset. Interestingly, the enrichment significance of most regulons remained unchanged after TP adjustment—they were either consistently identified or not identified (Fig. S3h). Only a small subset of regulons, including Mce3R, PhoP, FasR, and WhiB1, became significantly (adjusted *P* < .1) enriched after adjustment. These results are biologically consistent: PhoP is involved in pH regulation and has been shown to respond to cholesterol [28]. FasR regulates fatty acid biosynthesis [31]. WhiB1 is regulated by Rv2788, which can alter cell wall fatty acids [32]. In contrast, the Lsr2 regulon lost significance after TP adjustment. Given that Lsr2 functions as a nucleoid-associated protein involved in chromosome organization [33], its DE under cholesterol treatment may reflect nonspecific transcriptional changes rather than a targeted biological response. Together, these findings underscore that TP adjustment not only improves gene-level rankings but also enhances the sensitivity and specificity of enrichment-based analyses for biologically meaningful regulons.

### TP-adjusted DE analysis can capture low-TP but responsive genes

**Figure 2:**
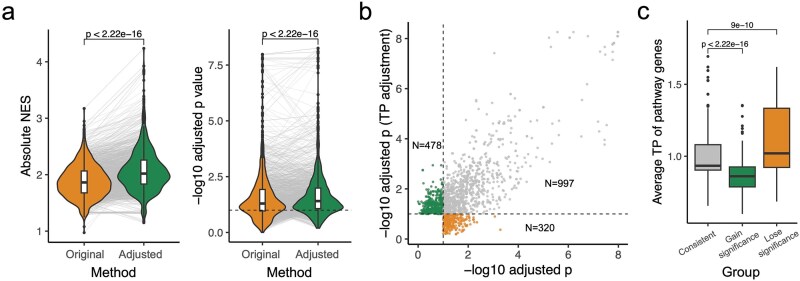
Regulon and pathway analysis based on DE analysis after TP adjustment. (a) Absolute NES (left panel) and statistical significance (adjusted *P*-value, right panel) of 197 gene pathways that surpassed the significance threshold (adjusted *P* < .1) for enrichment in the total 127 *Mtb* datasets (1795 pathway-dataset pairs in total) are both significantly increased after adjustment. Boxes show the median and IQR, whiskers represent the median ± 1.5 × IQR, and black dots indicate outliers. Each line represents a gene pathway in one RNA-seq dataset. Paired Wilcoxon *P*-values are shown. The horizontal black dashed line (right) represents adjusted *P*-value of 0.1. (b) Adjusted *P*-values of gene pathways before and after TP adjustment, where each dot represents a single pathway. Green dots (*N* = 478) indicate pathways with an adjusted *P*-value of gene pathways before and after TP adjustment, where ea*p* value < 0.1) after adjustment. Orange dots (*N* = 320) represent pathways that were significant (adjusted *P* < .1) before adjustment but became nonsignificant (adjusted *P* < .1) before adjustment but became nonsignificant [adjusted ays that were signit (adjusted *P* < .1) both before and after adjustment. (c) Compared to gene pathways that remained significant before and after adjustment (“consistent”), pathways that gained significance (adjusted *P* < .1) both before and after <0.1 after adjustment) exhibit significantly lower average TP values, whereas pathways that lost significance (adjusted *P* < 0.1 before adjustment and ≥0.1 after adjustment] have higher TP values. Boxes show the median and IQR, whiskers represent the median es, wh × IQR, and black dots indicate outliers. Wilcoxon test *P*-values are shown.

To determine whether the TP-adjusted strategy can improve the identification of new responsive gene pathways, particularly those with low TP, we also applied GSEA [7] to each *Mtb* RNA-seq dataset and then compared the identification of DE pathways before and after adjustment (Data S3). Across the 127 *Mtb* datasets, TP adjustment increased the overall absolute NES as well as the enrichment significance of the 1795 pathway-dataset pairs (including 197 unique gene pathways) that surpassed the significance threshold (adjusted *P* < .1) (Fig. 2a, Table S4).

Next, we tested whether adjusted DE levels could identify new responsive pathways. After adjustment, among the total 1795 pathway-dataset pairs, 478 pairs (26.6%) with lower TP gained statistical significance (“gain significance”, Fig. 2b and c, Fig. S4, Table S4). In contrast, 320 pairs (17.8%) in pathways with high TP were no longer significant after adjustment (“lose significance”, Fig. 2b and c, Fig. S4, Table S4). The remaining 997 pairs (55.5%) were significant before and after adjustment.

**Figure 3:**
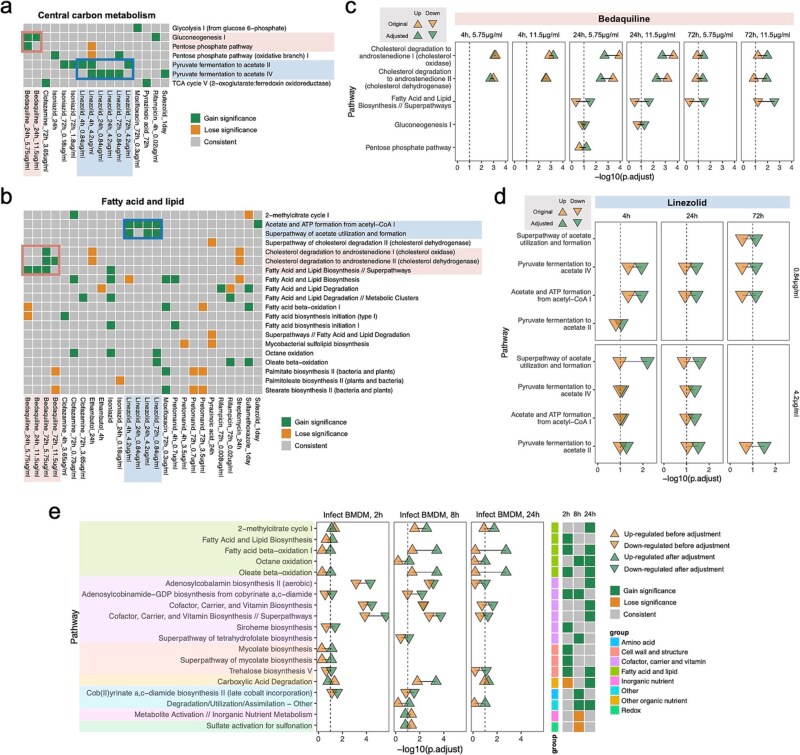
Metabolic responses of *Mtb* to antibiotics after TP adjustment. (a and b) Heatmaps showing the significance of central carbon metabolism (a), and fatty acid/lipid metabolism (b), pathways under antibiotic treatments. Green, orange, and grey rectangles represent “gain significance”, “lose significance”, and “consistent” pathways, respectively, where “consistent” pathways are both significant or both nonsignificant before and after TP adjustment. Highlighted pathways under bedaquiline and linezolid treatments are marked in red and blue, respectively. (c and d) Enrichment results of highlighted pathways in bedaquiline (c) and linezolid (d) treatments. Green and orange triangles indicate “gain significance” and “lose significance” pathways, respectively. Regular and inverted triangles denote upregulation and downregulation. (e) Enrichment results of pathways in the BMDM infection model. Green and orange triangles indicate “gain significance” and “lose significance” pathways, respectively, with regular and inverted triangles denoting upregulation and downregulation. The heatmap (right) shows significance changes, where green, orange, and grey rectangles represent “gain significance,” “lose significance,” and “consistent” pathways, respectively. Pathway groups are annotated in Table S5.

We then focused on characterizing the pathways that gained significance after adjustment. Because the bacterial metabolic state can influence antibiotic efficacy [34, 35], we first examined the response of metabolic pathways to antibiotics (Fig. 3a and b). Interestingly, after bedaquiline and linezolid exposure, we observed responses in central carbon and fatty acid/lipid metabolism pathways (Fig. 3a and b, Table S5) that were masked before TP adjustment. After 24 h of exposure to bedaquiline, the gluconeogenesis pathway was downregulated and the pentose phosphate pathway (PPP) was upregulated (Fig. 3c). This correlates with the recent observation that with bedaquiline exposure, *Mtb* reroutes acetate, a metabolite from the degradation of even chain fatty acids, from gluconeogenesis into the PPP [36]. Although no cholesterol was added to the media, genes related to cholesterol degradation were induced after 72 h of bedaquiline treatment, consistent with the results at earlier time points (Fig. 3c). Intriguingly, *Mtb* exhibited hypersensitivity to bedaquiline when grown in media supplemented with cholesterol [37], suggesting a potential interplay between bedaquiline responses and cholesterol degradation. For linezolid, TP-adjusted DE analysis revealed reduced acetate production and utilization across different drug concentrations and treatment times, although before TP adjustment, these pathways were identified only in specific concentrations and exposure times (Fig. 3d).

To determine whether the TP adjustment could also aid in identifying transcriptional responses to host-related conditions, we applied the TP-adjusted strategy to RNA-seq results from three experiments involving *Mtb* infections of Bone Marrow Derived Macrophages (BMDM) and compared the results before and after adjustment. The TP-adjusted results identified novel *Mtb* pathways responsive to macrophage infection, including pathways related to fatty acid/lipid metabolism, cofactor/carrier/vitamin biosynthesis, and cell wall structure (Fig. 3e, Table S5). The enhanced synthesis and utilization of fatty acids and lipids reflects the *Mtb* preference for fatty acids as a carbon source within host cells [38, 39], including their cholesterol-rich lipid droplets [40]. Synthesis of adenosylcobalamin, or coenzyme B12, and siroheme, which is structurally and biosynthetically related to the corrin ring of vitamin B12, were both down-regulated during BMDM infection (Fig. 3e), which may be related to the uptake of host vitamin B12 [41]. Increased mycolate synthesis during BMDM infection results in a thicker cell wall (Fig. 3e) that may promote intracellular survival. Synthesis of trehalose, another essential component of mycobacterial cell wall, was reduced in both BMDM infection and in a hypoxia model *in vitro* (Fig. 3e, S5a). Trehalose synthesis was up-regulated in multiple antibiotic treatments (Fig. S5a), suggesting a potential effect on drug susceptibility [42].

### Transcriptional plasticity adjustment is applicable to new transcriptomic data and other bacteria

**Figure 4:**
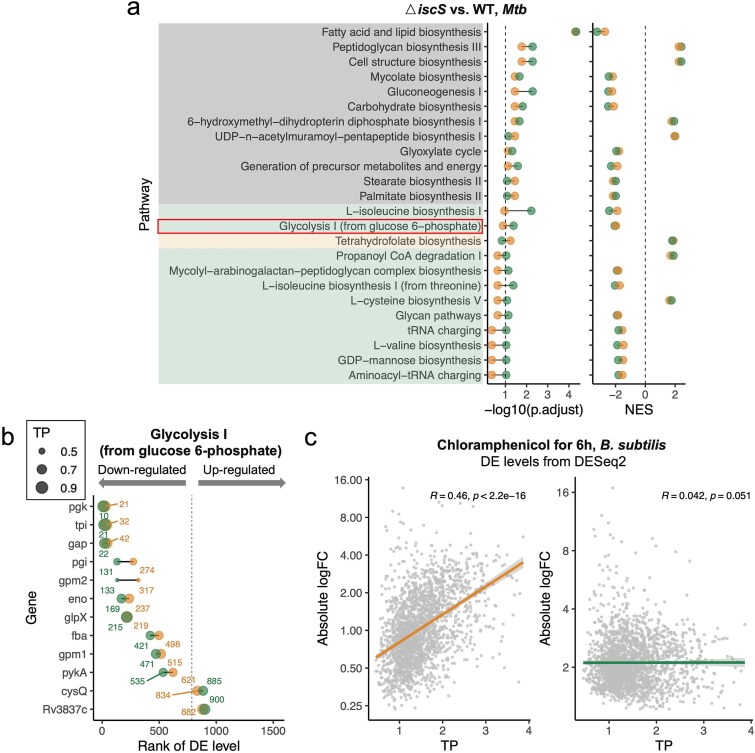
Adjusting DE levels in new RNA-seq datasets. (a) Adjusted *P*-values (left) and NES (right) of gene pathways enriched in Δ*iscS* strains in GSEA. Green, orange, and Grey rectangles represent pathways with “gain significance”, “lose significance”, and “consistent” status, respectively. Orange dots indicate values before adjustment, while green dots represent values after adjustment. Grey dashed lines denote an adjusted *P*-value of 0.1 (left) and an NES of 0 (right). The glycolysis pathway is highlighted (red box). (b) the ranking of glycolysis-related genes (highlighted in Fig. 4a) improved after adjustment. Orange dots indicate ranks before adjustment, and green dots indicate ranks after adjustment. Numbers represent gene ranks, while dot size corresponds to TP values. Dots to the left of the dashed line represent down-regulated genes, where smaller rank numbers indicate higher ranks among downregulated genes. Dots to the right represent up-regulated genes, where larger ranking numbers indicate higher ranks among upregulated genes. (c) Absolute log₂ FC values are significantly correlated with TP before adjustment (left) but no longer correlated after adjustment (right) for genes with an adjusted *P* < .05, obtained using the DESeq2 method in *B. Subtilis* after chloramphenicol treatment for 6 h. Spearman’s correlation coefficients and the corresponding *P*-values are shown.

To further test the effectiveness of the TP adjustment, we used it to analyze three recently released RNA-seq datasets [15], including two knock-out mutants and one chemical treatment (Figs S1e–g, Table S6). Significant correlations between DE levels and TP for both up-regulated and down-regulated genes in the three datasets were eliminated after adjustment (Figs S6a–c). We then focused on the dataset of *△iscS* because it included metabolomic data that could validate the TP-adjusted DE results [21]. *iscS* encodes cysteine desulfurase i.e. involved in iron–sulfur biogenesis [21]. GSEA analysis based on the TP-adjusted DE levels in the *△iscS* strain identified down-regulation of the synthesis pathways for multiple amino acids, which were not identified before TP adjustment (Fig. 4a, Table S7). The glycolysis pathway was also down-regulated in *△iscS* strains (Fig. 4a), which was consistent with the reduced presence of glycolytic intermediates measured by quantitative liquid chromatography-mass spectrometry (LC–MS/MS) [21]. The DE levels in both down- and up-regulated genes involved in glycolysis ranked higher after adjustment (Fig. 4a and b), especially the low TP genes *pgi* and *gpm2* TP (Fig. 4b). TP adjustment also highlighted the down-regulation of fumarate hydratase *fum* in the *△iscS* dataset (Fig. S6d), consistent with the increase in fumarate and the decrease in downstream metabolite malate that was seen on LC–MS/MS [21].

Finally, to evaluate whether the TP-adjustment strategy could be applied to other bacterial species, we obtained RNA-seq datasets from the Gram positive bacterium B. subtilis and the Gram negative P. aeruginosa and measured the TP of their genes using the same approach used for *Mtb* and E. coli (Materials and Methods) [43, 44]. Then, to validate the TP adjustment strategy for each species, we collected newly generated RNA-seq datasets from chloramphenicol treatment of B. subtilis [45] and phenylalanine arginine-olnaphthylamide (PaβN) treatment of P. aeruginosa [46]. As expected, the DE levels in the validation datasets showed significant correlations with TP, which were eliminated after TP adjustment (Fig. 4c, Fig. S6e). To make TP adjustment accessible and easy to use, we provide a streamlined, one-click method for researchers to apply TP adjustment to DE analysis (Fig. S7).

## Discussion

In this study, we evaluated the impact of TP on standard DE analyses and introduced a TP-adjustment approach to mitigate its effects. By applying LOESS adjustment, we corrected for the correlation between TP and DE levels, which improved the detection of bacterial responses in both regulons and gene pathways. The adjustment also increased the statistical significance and enrichment scores of DEG, enabling the identification of previously overlooked response pathways. Furthermore, we showed that the adjustment strategy can correct the correlation between TP and DE levels in other bacterial species and can be used with RNA-seq data collected and quantified with different methodologies.

Adjusting the DE levels based on each gene’s TP may allow the identification of new metabolic pathways that respond to antibiotic exposure, which could facilitate the understanding of drug susceptibility. For example, studies have suggested that steroids may enhance the anti-*Mtb* efficacy of antibiotics such as bedaquiline [37, 47]. The TP-adjustment strategy expanded the panel of DE genes responding to bedaquiline treatment and highlighted the simultaneous activation of cholesterol degradation and suppression of lipid biosynthesis (Fig. 3c). When cholesterol and other steroids were present at high concentrations, up-regulated cholesterol degradation and decreased lipid synthesis could cause *Mtb* to accumulate the toxic intermediate propionyl-CoA, leading to metabolic intoxication in addition to bedaquiline’s direct inhibition of ATP biosynthesis [48]. In contrast, linezolid suppressed the expression of cholesterol degradation-related genes (Fig. S5b) and was less effective at killing *Mtb* when cholesterol was present [37]. Together, these results imply that antibiotics can induce specific metabolic dysregulations that interfere with bacterial killing, and our TP-aware DE analysis can help identify the key metabolic pathways involved.

Some pathways that were significantly enriched with standard DE analysis became nonsignificant after adjustment, particularly pathways containing high-TP genes (Fig. 2c, Fig. 3, Fig. S4). Although these genes or pathways responded to the experimental conditions, the response was likely not specific for each condition. As an example, the responses of the high TP DosR regulon across various conditions (Fig. S2b and c) [15] decreased in significance after TP adjustment (Fig. S5c) suggesting that these DosR responses were not condition-specific. In contrast, the response of the DosR regulon to a dormant state remained significant even after adjustment, (Fig. S5c), suggesting a specific response. Another notable example is the phthiocerol dimycocerosate (PDIM) biosynthetic pathway (Fig. S8a), which GSEA analysis became statistically insignificant after TP adjustment across 23 experiments (Fig. S8b). PDIM is a critical virulence factor known to facilitate *Mtb* immune evasion [49, 50]. However, its biosynthesis is metabolically costly, particularly in axenic culture conditions, which drives frequent loss-of-function mutations in these pathways in the absence of host stress or host-mimicking carbon sources [51, 52]. A closer inspection of the dataset revealed that most instances of PDIM pathway significance loss occurred under antibiotic treatment conditions (Fig. S8b). Interestingly, a recent large-scale chemical-genetic screen found little to no direct connection between PDIM biosynthesis and antibiotic susceptibility [53]. Combining the above evidence and our analysis, we speculate that the functional associations between changes in PDIM gene expression and environmental cues are likely overestimated across studies. While we posit that TP-adjustment may mitigate such overestimation in pathway analysis, full validation would require future experimental studies.

At present, large-scale RNA-seq datasets for estimating gene TP are available for only a few bacteria, primarily model organisms such as E. coli and well-studied bacteria such as *Mtb*. The availability of extensive transcriptomic data is crucial for accurately assessing TP, as it requires diverse experimental conditions to capture the full range of gene expression variability. For many clinically or environmentally important bacterial species, large-scale transcriptomic datasets remain scarce. In addition, RNA-seq studies are often limited to specific experimental conditions or disease contexts, which don’t provide an adequate representation of TP. Expanding RNA-seq datasets across a broader range of bacterial species would not only enhance TP estimation but also improve the robustness of TP-aware DE analysis in these bacteria. Applying TP-based approaches to emerging multidrug-resistant pathogens, such as Acinetobacter baumannii and Klebsiella pneumoniae*,* could perhaps uncover new regulatory mechanisms underlying antibiotic resistance and virulence [54]. Similarly, for environmental bacteria with complex regulatory networks, such as those involved in biogeochemical cycling (*Pseudomonas spp.* or *Cyanobacteria*), TP-adjusted DE analysis could reveal novel adaptive responses to changing environmental conditions. Additionally, the application of single-cell RNA sequencing technology [55, 56] to bacterial transcriptomics may provide deeper insights into cell-to-cell variability, and TP estimation could be important for evaluating the significance of the differences.

In summary, we present a novel strategy that normalizes transcriptomic data to adjust for inherent transcriptional constraints, specifically TP, when analyzing DE results. This approach allows for a more accurate assessment of DE responses and facilitates the identification of previously overlooked bacterial responses across diverse experimental conditions. Future research can expand this strategy to reveal uncharacterized transcriptional adaptations and their functional implications for bacterial physiology and pathogenesis.

## Materials and methods

### Curation of existing transcriptomic, proteomic, and regulon data

We utilized an integrated RNA-seq dataset of M. tuberculosis H37Rv from our previous work [15], which included the expression levels of 3891 *Mtb* genes across 894 RNA-seq samples. To perform DE analysis, we excluded samples lacking replicates or corresponding controls, ultimately retaining 664 samples for analysis. Next, these samples were grouped into 127 contrasts based on the descriptions in their corresponding articles, with each contrast containing both experimental replicates and corresponding control samples (Table S1). For E. coli K-12 MG1655, we included 275 RNA-seq samples from the iModulon database (https://imodulondb.org/dataset.html?organism=e_coli&dataset=precise1k) and grouped them into 111 contrasts based on the original data annotations (Table S1). Notably, some control samples were repeatedly included in the DE analysis, as certain datasets contained multiple treatment conditions. Additionally, integrated transcriptomic data comprising 708 RNA-seq samples of B. subtilis (strain 168) and 820 RNA-seq samples of P. aeruginosa (PAO1) were obtained from the iModulon database for TP measurement (Table S1). *Mtb* regulon data were identified by Yoo et al [57]. The genes belonging to the DosR and Mce3R regulons are listed in Fig. S2a. A total of 35 *Mtb* regulons and their corresponding genes are listed in Table S3. Regulon genes without TP measurements were excluded from the analysis.

### Estimation of transcriptional plasticity

TP measurements for *Mtb* genes were obtained from our previous work (Table S2) [15]. To estimate TP for E. coli genes, we applied a similar data processing strategy. First, read count data for 4355 genes across 589 RNA-seq samples were collected from the iModulon database [44]. Genes shorter than 150 bp or those not expressed in any RNA-seq sample were excluded. Read counts were then normalized using the Trimmed Mean of M-values (TMM) method to account for variations in library size [58]. RPKM values were subsequently calculated using the R package *edgeR* (version 3.30.3) [59]. The Shannon index (SI) of RPKMs across RNA-seq samples was measured for each gene using the R package *vegan* (version 2.5-7). Genes in the lowest 1% SI or those not expressed in more than 1% of the total samples were excluded. Additionally, we removed samples with a high proportion of nonexpressing genes (>5% of total genes). A total of 4185 genes and 582 samples were included for TP estimation. To estimate TP, residuals from LOESS regression of the standard deviation (SD) against the mean log₂(RPKM+1) values were added to the global average of the LOESS-fitted SD measures across all included genes. LOESS regression was performed using the R package stats (version 4.0.2) with parameters span = 0.7 and degree = 1. The TP estimation process for B. subtilis and P. aeruginosa followed the same methodology as for E. coli. TP measurements for genes in E. coli*,* B. subtilis, and P. aeruginosa are listed in Table S2.

### TP adjustment for DE analysis

For each contrast in RNA-seq datasets, samples were grouped into treatment condition and control condition. Limma method from R package *limma* (version 3.44.3) was used to generate DE levels (log₂ FC) and corresponding *P*-values for the expression of genes in the treatment condition compared to the control condition [3]. Genes with adjusted *P* < .05 were identified as DEGs and were retained in further adjustment. We identified 294 140 DE events of *Mtb* genes and 192 695 DE events of E. coli genes in all RNA-seq contrasts (one DE event refers to one DEG identified in one dataset), and their DE levels are listed in Data S1 (*Mtb*) and Data S2 (E. coli), respectively. To adjust DE levels, the global trend between TP and absolute DE levels of DEGs in each contrast was estimated by logarithmic LOESS regression using the R package *stats* (version 4.0.2; span = 0.5, degree = 1). Logarithmic LOESS regression was used because both the TP and log₂ FC values did not obey a normal distribution. This could lead to a large bias in LOESS fitting for genes with high TP or high log 2FC because the number of these genes were sparse, but logarithmic processing could bring the data closer to a normal distribution. The reasoning for adjusting the log_2_ FC is to use the residual between the log₂ FC and the global trend between TP and log₂ FC as a TP-independent log₂ FC:


$$ {e}_i={Y}_i-{\hat{Y}}_i $$

where *i* represents the i^th^ gene, and *e_i_* is the residual for the i^th^ gene. *Y_i_* is the real absolute log_10_ (log₂ FC) (corresponding to logarithmic LOESS fitting) and ${\hat{Y}}_i$ is the absolute log_10_ (log₂ FC) predicted by log_10_ (TP) according to the LOESS fitting. The log₂ FC residual was then added to the logarithmic mean value of LOESS fitted log₂ FC:


$$ {\displaystyle \begin{array}{c}{\left| Adjusted\ {\log}_2\ FC\right|}_i={10}^{e_i}+{10}^{\frac{\sum_{i=1}^N{\hat{Y}}_i}{N}}\end{array}} $$

where *N* represented the total number of genes. Adjustment was performed separately for up-regulated and down-regulated genes.

### Gene set enrichment analysis

GSEA was performed for each RNA-seq analysis using log₂ FC or adjusted log₂ FC measures of DEGs with the R package clusterProfiler (version 3.16.1; parameters: eps = 1e-10, minGSSize = 1). Gene pathway annotations for *Mtb* genes were obtained from BioCyc (https://www.biocyc.org) and are listed in Table S8. Pathways with an adjusted *P* < .1 were regarded as significant. Gene pathways were manually categorized based on their functional groups (Table S5). Gene pathways with an adjusted *P*-value pathways with an adjusted i < 0.1 after adjustment were classified as “gain significance”. Pathways with an adjusted *P* < .1 before adjustment but ≥0.1 after adjustment were classified as “lose significance.” Pathways with an adjusted *P* < .1 both before and after adjustment were categorized as “consistent”.

### Statistical analysis

Spearman’s correlations coefficients and the corresponding *P*-values were shown in Fig. 4c, Figs S1c–g and S6a–c, S6e. Paired Wilcox tests were performed in Fig. 1h, Fig. 2a and Fig. S3g. Unpaired Wilcox tests were performed in Fig. 2c and Fig. S2c.

**Key Points**

- Transcriptional plasticity (TP), an intrinsic gene property that defines the expected range of expression variation across conditions, can introduce bias in RNA-seq differential expression (DE) analyses.
- We developed a simple yet effective TP-adjustment strategy that reveals novel, biologically meaningful bacterial responses overlooked by traditional approaches.
- We validated the generalizability of our framework using datasets from other bacterial species and facilitated broader adoption by providing a user-friendly, one-click R script.

## Supplementary Material

Figure_S1_bbaf557

Figure_S2_bbaf557

Figure_S3_bbaf557

Figure_S4_bbaf557

Figure_S5_bbaf557

Figure_S6_bbaf557

Figure_S7_bbaf557

Figure_S8_bbaf557

Table_S1_bbaf557

Table_S2_bbaf557

Table_S3_bbaf557

Table_S4_bbaf557

Table_S5_bbaf557

Table_S6_bbaf557

Table_S7_bbaf557

Table_S8_bbaf557

## Data Availability

RNA-seq data included in this study are listed in Table S1. TP measurements for *Mtb*, E. coli, B. subtilis and P. aeruginosa genes are available in Table S2. DE analysis results are provided in Data S1 and S2. GSEA results are shown in Data S3 and Table S4. Other source data and analyzing code are provided on GitHub: https://github.com/ChengBEI-FDU/Adjust_differential_expression.
