# Supplementary figures and images for: A transcriptional plasticity-aware framework for RNA-seq differential expression analysis
Source: Brief Bioinform. 2025 Oct 20;26(5):bbaf557. doi: 10.1093/bib/bbaf557 (PMC12536879; doi:10.1093/bib/bbaf557)

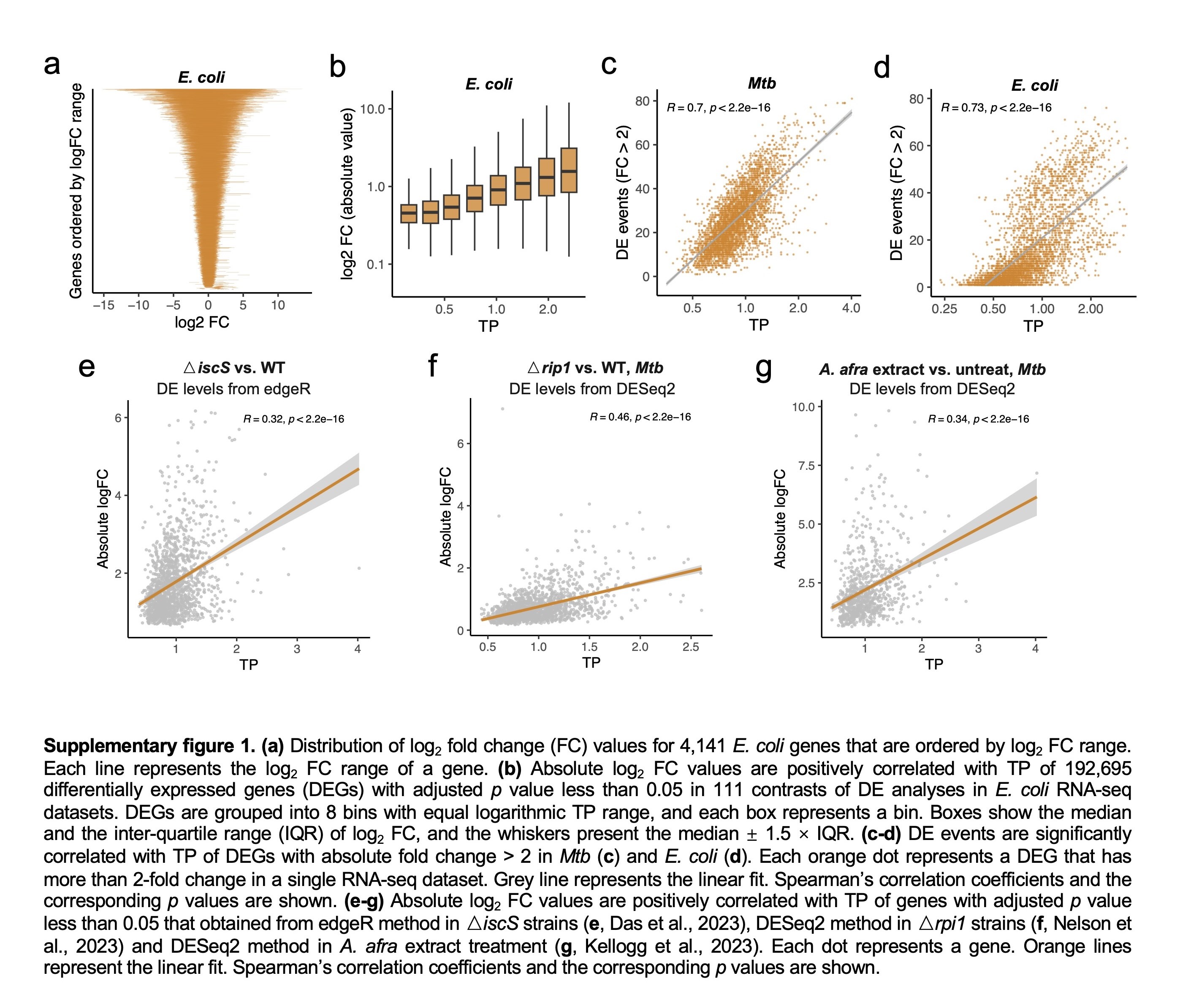

Supplement: Figure_S1_bbaf557 [file figure_s1_bbaf557.jpeg]

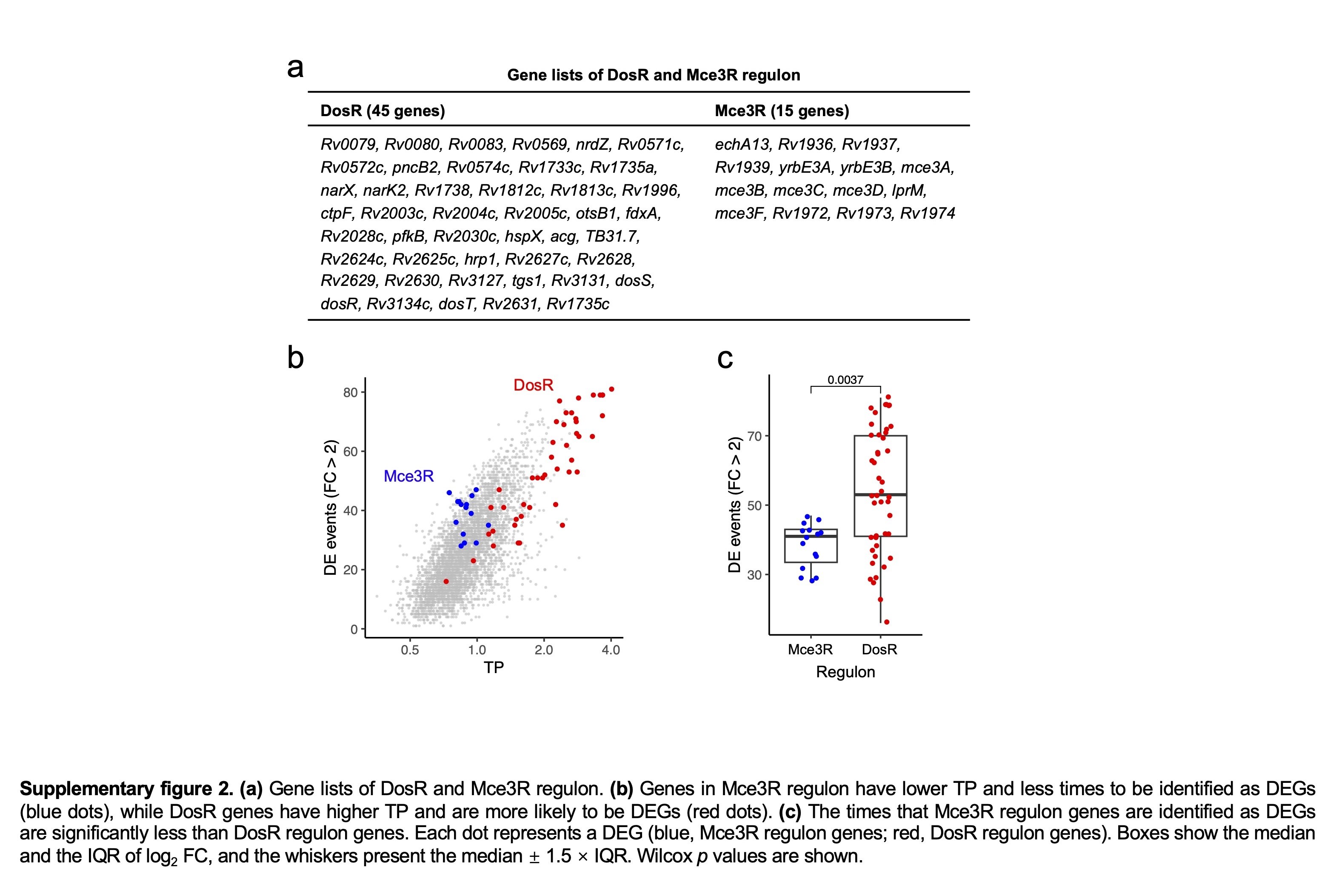

Supplement: Figure_S2_bbaf557 [file figure_s2_bbaf557.jpeg]

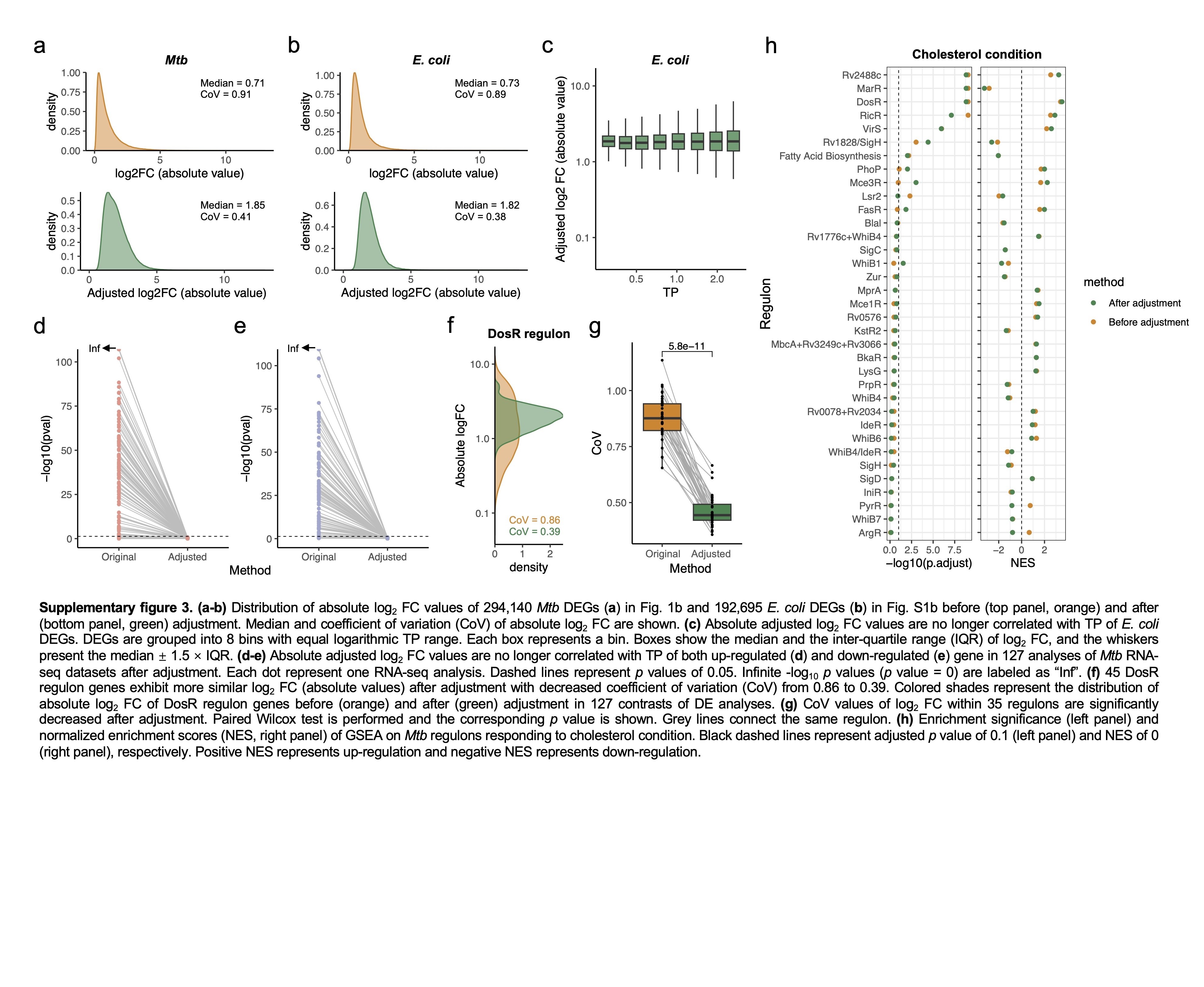

Supplement: Figure_S3_bbaf557 [file figure_s3_bbaf557.jpeg]

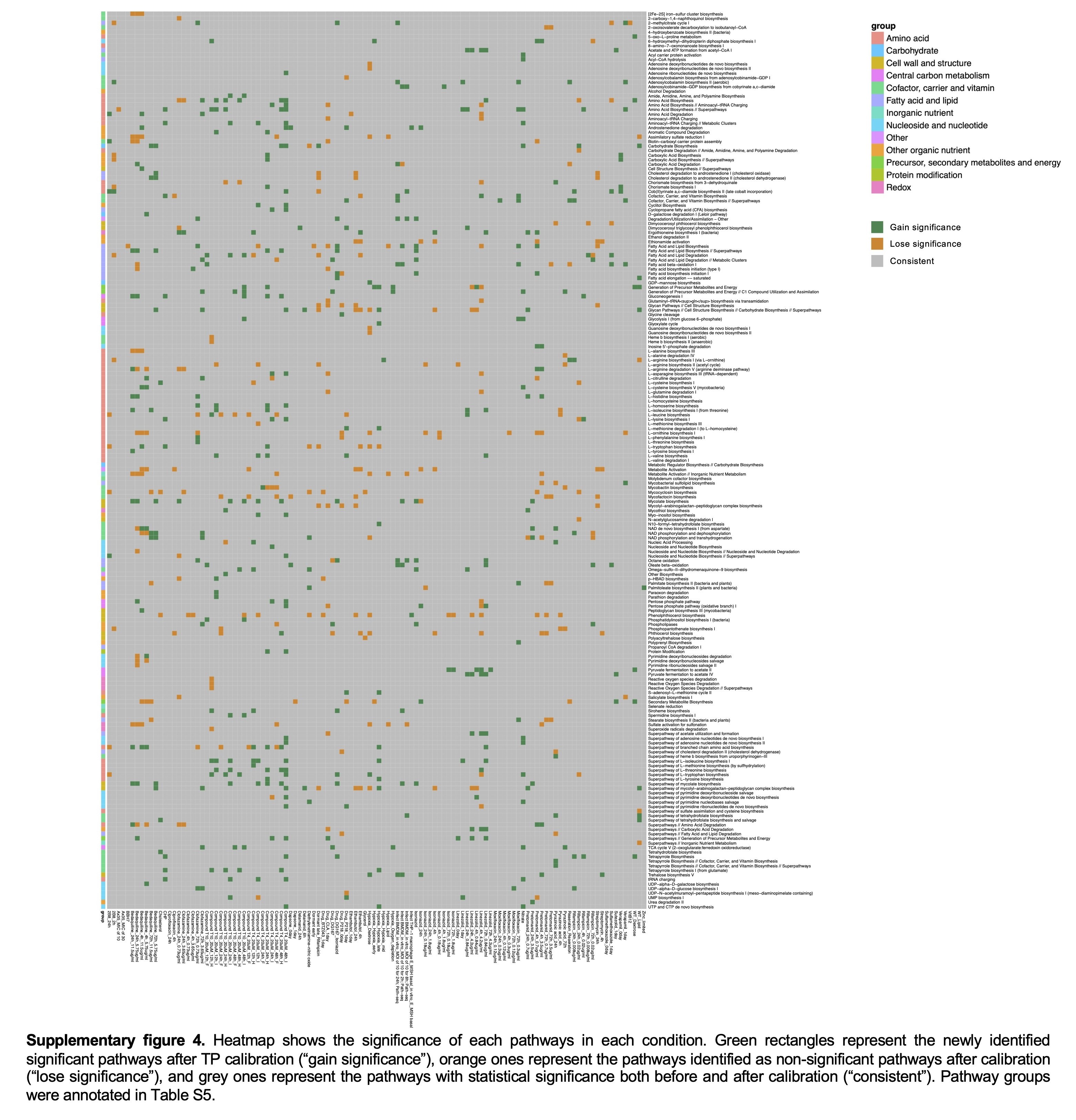

Supplement: Figure_S4_bbaf557 [file figure_s4_bbaf557.jpeg]

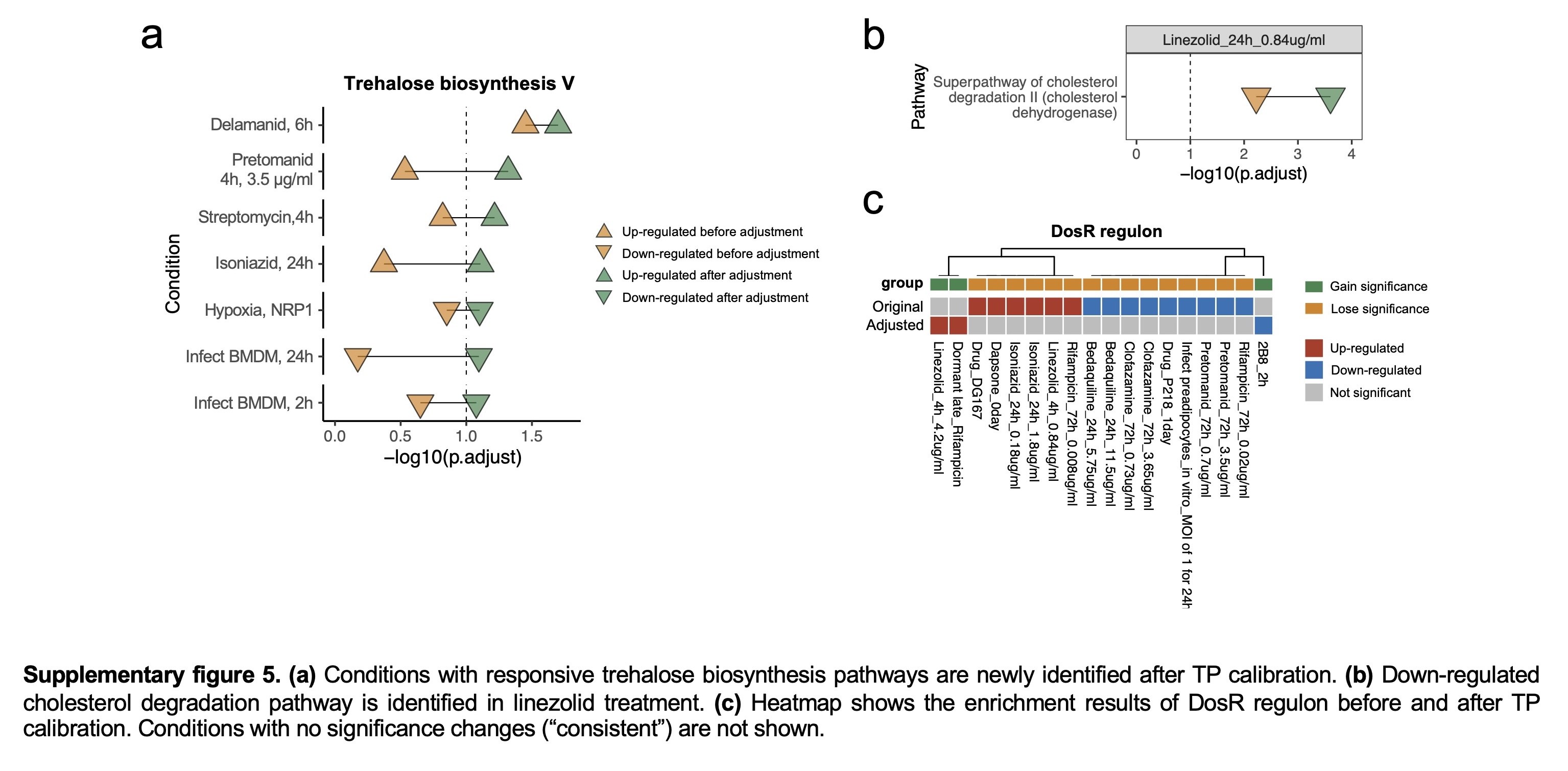

Supplement: Figure_S5_bbaf557 [file figure_s5_bbaf557.jpeg]

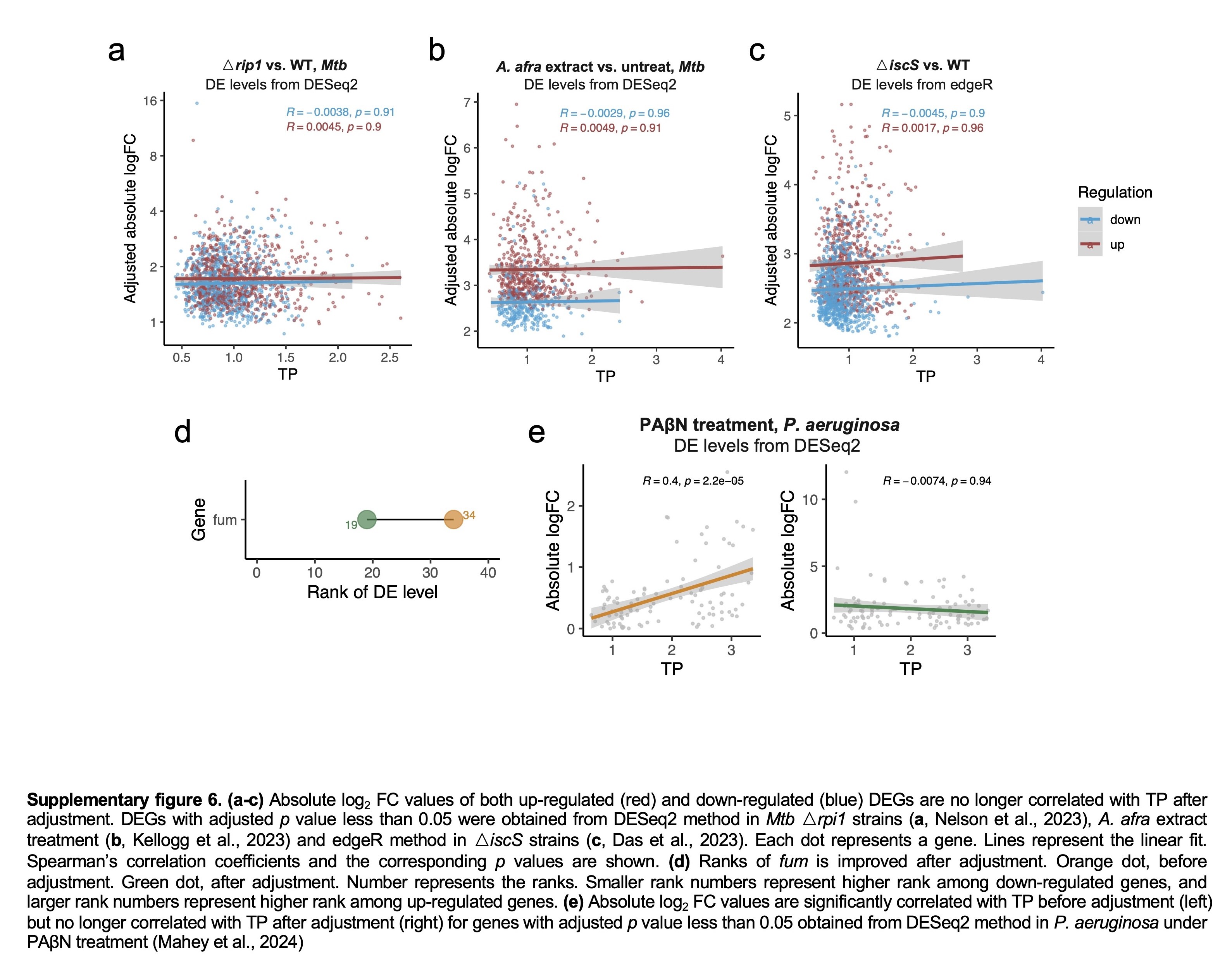

Supplement: Figure_S6_bbaf557 [file figure_s6_bbaf557.jpeg]

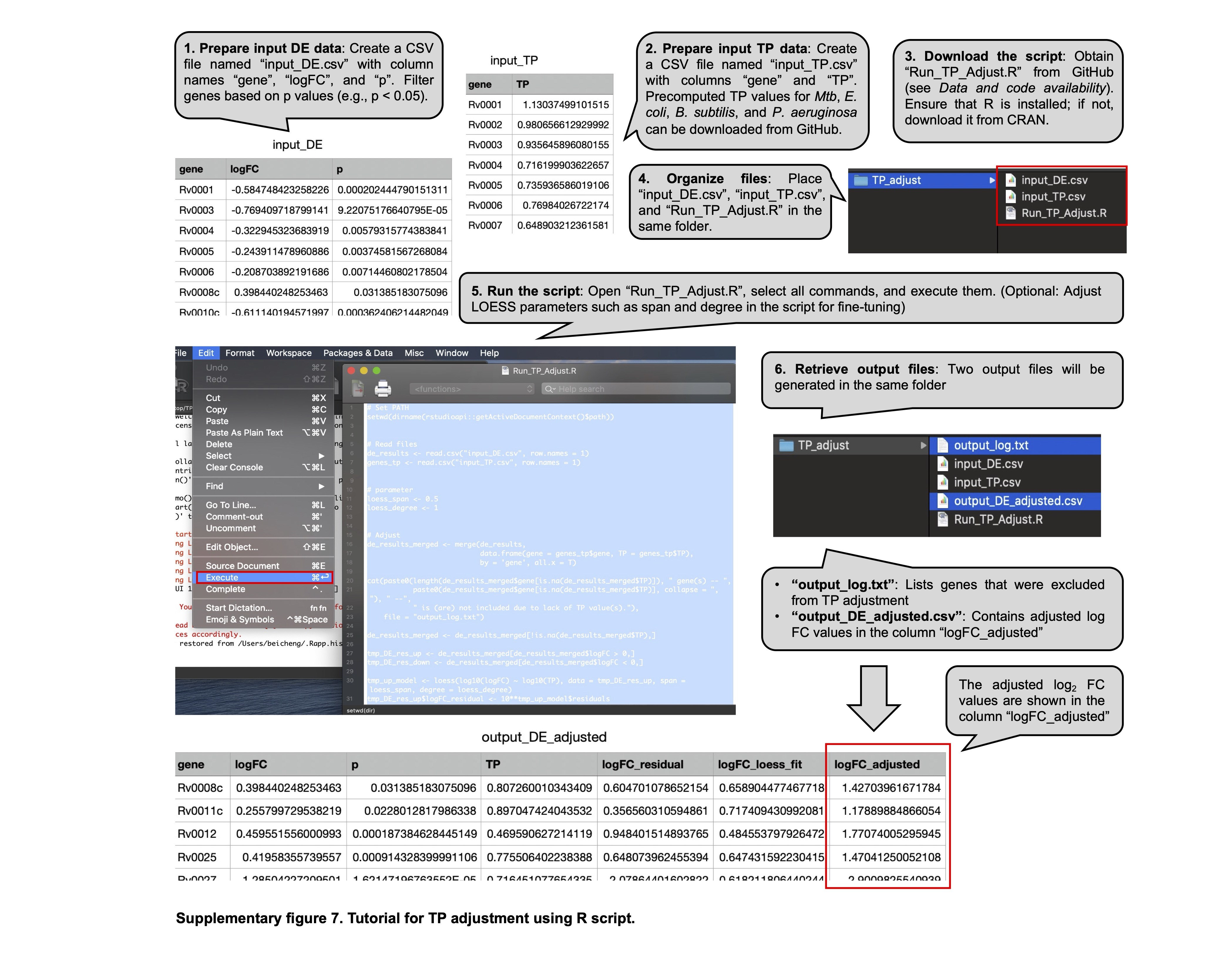

Supplement: Figure_S7_bbaf557 [file figure_s7_bbaf557.jpeg]

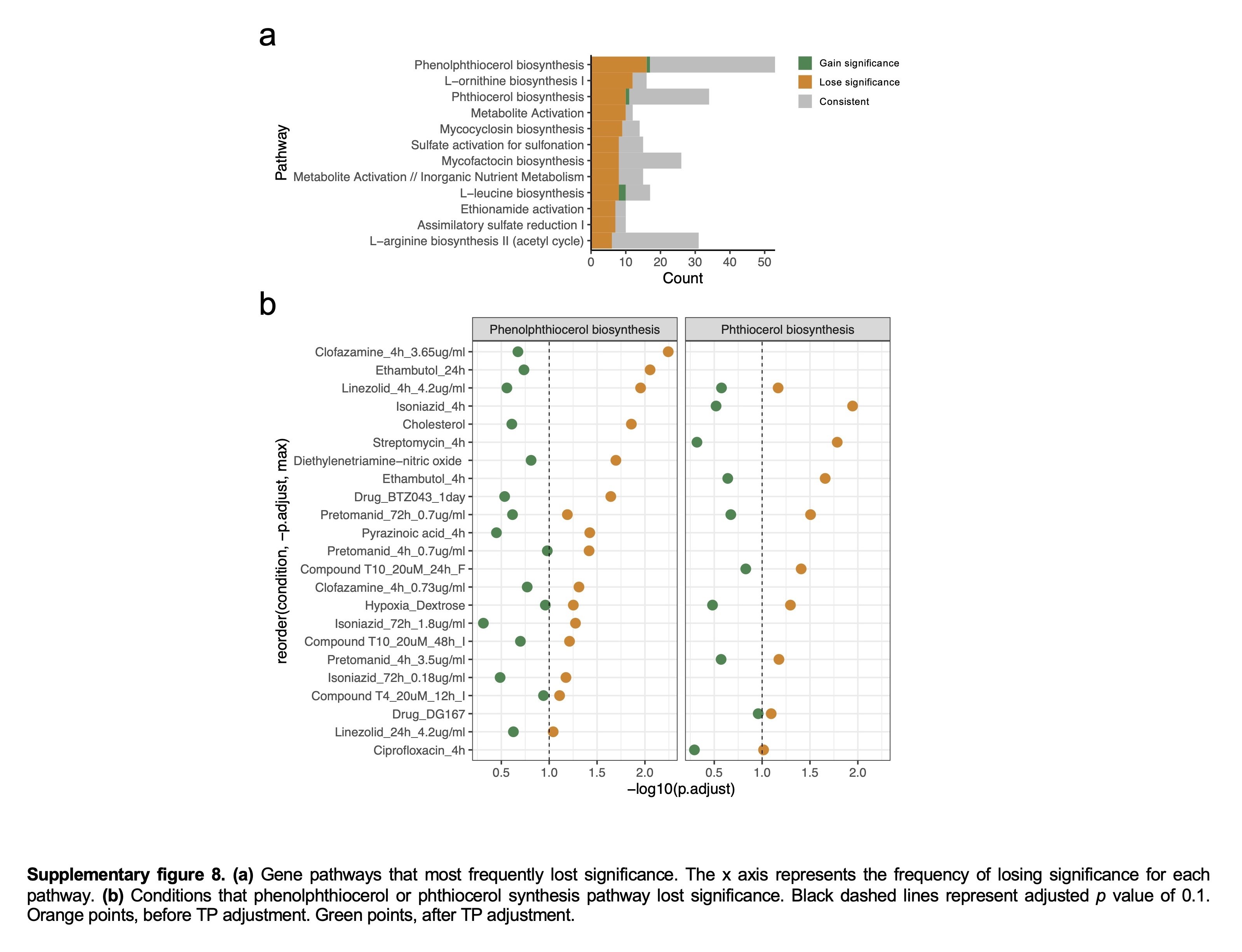

Supplement: Figure_S8_bbaf557 [file figure_s8_bbaf557.jpeg]
